# Clinical Presentation and Histopathological Characteristics of Sporotrichosis

**DOI:** 10.1111/jocd.70059

**Published:** 2025-03-14

**Authors:** Xiaohong Zhao, Panpan Li, Jianxun Yang

**Affiliations:** ^1^ Department of Dermatology The 2nd Hospital of Harbin Medical University Harbin China

**Keywords:** clinical manifestations, fungal infections, histopathology, inflammatory infiltration, sporotrichosis

## Abstract

**Objective:**

Sporotrichosis is the most prevalent deep mycosis worldwide. The differential diagnosis of its clinical symptoms and histopathological features presents considerable complexity. A thorough understanding of the clinical manifestations and pathological characteristics of sporotrichosis is crucial for its accurate clinical diagnosis.

**Methods:**

This study involved a retrospective analysis of cases diagnosed with sporotrichosis at our hospital between April 2017 and April 2019, summarizing their clinical and histopathological characteristics.

**Results:**

Among the 323 patients included in this study, a higher prevalence was observed in females. The disease predominantly affected middle‐aged and elderly patients, aged between 41 and 67 years, with a higher incidence noted in winter and spring. Among the 323 patients, 170 patients (52.7%) presented with the fixed pattern, 128 (40%) with the lymphatic type, 24 (7%) with the special type, and one patient with the cutaneous‐disseminated type. There were no cases of the systemic type. Histopathological findings included diffuse infectious granulomas and inflammatory infiltration, primarily characterized by diffuse and non‐specific mixed inflammatory cell infiltration (without granulomas). The granulomas were primarily tuberculoid and purulent, with a lesser occurrence of granulomas exhibiting a three‐zone structure, which is typically uncommon. An increase in lymphocytes, plasmacytes, and neutrophils was noted as one of the pathological features of the disease.

**Conclusion:**

In this region, sporotrichosis was more prevalent among female patients compared to male patients, with the fixed pattern being more common than the lymphatic type. Histopathological manifestations included diffuse infectious granulomas, with inflammatory infiltration primarily characterized by diffuse infiltration and non‐specific mixed inflammatory cell infiltration (without granulomas).

## 1 Introduction

Sporotrichosis is a chronic granulomatous zoonosis caused by infection with pathogenic bacteria of the *Sporothrix* complex, predominantly found in subtropical regions [1]. This condition was first detected in 1898 by Schenck in the United States, who also isolated the pathogenic bacteria. *Sporothrix* is a dimorphic fungus that exists as mycelia in nature at room temperature and as yeast in vivo, forming small blastospores within tissues. In recent years, there has been a significant increase in reported cases of sporotrichosis, particularly in northeast China, with incomplete statistics indicating over 2000 cases.

The clinical manifestations of sporotrichosis range from mild to severe and can be categorized into four types: cutaneous‐fixed, cutaneous‐lymphatic, cutaneous‐disseminated, and extracutaneous. To achieve a more comprehensive understanding of sporotrichosis, Professor Li Fuqiu further subdivided the fixed type into ulcerative, papular nodule, and squamous patch subtypes. Additionally, special forms resembling acne, rosacea, and herpes zoster have been discovered [2].

The histopathology of sporotrichosis is generally considered non‐specific; however, it remains valuable for inferential diagnosis due to its high sensitivity [3]. Through extensive analysis of pathological data, the inflammatory infiltration in sporotrichosis has been classified into non‐specific inflammatory infiltration, poorly formed granulomas, and well‐formed granulomas. Granulomas are further categorized into tuberculoid, purulent, three‐zone, palisading types, and so on [4]. A literature review indicates that the chronic nature of sporotrichosis, both in clinical manifestations and histopathology, is influenced by factors like the immune status of the host, the virulence and heat tolerance of the pathogenic fungi, the inoculum size, the depth of trauma, and the duration of the disease [5, 6]. There is a correlation between the severity of clinical manifestations and the immune status of the body, with poorer immunity associated with more severe clinical symptoms. Consequently, the more severe the clinical manifestations, the higher the fungal load.

To enhance the understanding of sporotrichosis and advance clinical diagnosis and treatment, a retrospective analysis was conducted on the clinical manifestations and histopathology of 323 patients diagnosed with sporotrichosis at the Department of Dermatology, the Second Affiliated Hospital of Harbin Medical University, from April 2017 to April 2019.

## 2 Research Participants and Methods

### 2.1 Research Participants

Inclusion criteria: Patients diagnosed with sporotrichosis at the Department of Dermatology, the Second Affiliated Hospital of Harbin Medical University, from April 2017 to April 2019. Diagnostic criteria: Patients exhibiting various clinical types of skin lesions of sporotrichosis, a positive result on fungal culture, and confirmation through histopathological reports of infectious granulomas.

### 2.2 Cases Data

The names, sexes, ages, medical histories, and clinical types of the enrolled patients were documented. Additional information, such as occupations, history of trauma, medications, treatment duration, treatment outcomes, was obtained through telephone follow‐up interviews.

### 2.3 Hematoxylin–Eosin (HE) Staining of the Histopathological Sections of Skin Lesions

Paraffin blocks containing tissues from 323 patients were individually sliced and fixed. Tissue sections were then deparaffinized and hydrated. Subsequently, the sections were stained with hematoxylin, fixed with 1% ethanol hydrochloride after rinsing, and turned blue with bluing solution. Eosin solution was used for additional staining. Following water washing, the sections underwent sequential dehydration and clearing in 95% ethanol, absolute ethanol, dimethylbenzene I, and dimethylbenzene II. Finally, the sections were sealed with neutral balsam. Pathological sections from all 323 cases were examined under a microscope, and changes in the epidermis and dermis were observed and recorded. The pathological characteristics of sporotrichosis were then subjected to statistical analysis.

### 2.4 Fungal Culture

The tissue specimens were inoculated on Sabouraud dextrose agar (SDA) medium and placed in an incubator for cultivation. After 1 week, colonies with a diameter of 0.5 cm were observed, appearing milky white or light brown, with a membranous and slightly moist surface. By the second week, colonies grew to approximately 2 cm in diameter, characterized by a peripheral bulge, central depression, and folds. A dark brown coloration indicated a positive result for *Sporothrix*.

### 2.5 Statistical Methods

Statistical analysis of the basic characteristics, clinical manifestations, and histopathological results of the patients was conducted using EXCEL 2016 and SPSS 26.0 software.

## 3 Results

### 3.1 General Data

Among the 323 patients, there were 102 males and 221 females, resulting in a male‐to‐female ratio of 1:2.17 (*χ* ^2^ = 0.016, *p* = 0.905, indicating no statistical significance). The age of onset ranged from 1 to 90 years, with an average onset age of 51.7 years. The disease predominantly affected middle‐aged and elderly patients aged 41–67 years, comprising 62.0% of all cases. Among the 185 patients who underwent follow‐up visits, most resided in rural areas. Seventy patients reported a history of trauma, predominantly due to puncture wounds from corn stalks, flowers, and branches, while others had exposure to soil, corn stalks, flowers, and plants. Occupationally, 141 patients were farmers, 10 were students, 24 were unemployed or retired, and 10 were classified as other workers.

Of the 323 patients, 123 (38.2%) visited the hospital in spring, 63 (19.6%) in summer, 43 (13.4%) in autumn, and 93 (28.8%) in winter. There were statistically significant differences in the seasons when patients visited hospitals (*χ* ^2^ = 9.938, *p* = 0.004). The disease course, referring to the duration of disease, ranged from a minimum of 20 days to a maximum of 5 years, with an average duration of (3.2 ± 1.82) months. The relationship between disease duration and the number of patients is presented in Table 1.

**TABLE 1:** Course of disease and number of patients.

Disease course (months/years)	Number of patients (*n*)	Proportion of total patients (%)
≤ 3 months	124	38.4
3 to ≤ 6 months	104	32.2
6 to ≤ 9 months	34	10.6
9 to ≤ 1 year	52	16.1
> 1 year	9	2.7
Total	323	100

*Note: p* = 0.025, significant correlation.

### 3.2 Clinical Manifestations

#### 3.2.1 Clinical Classification

**FIGURE 1:**
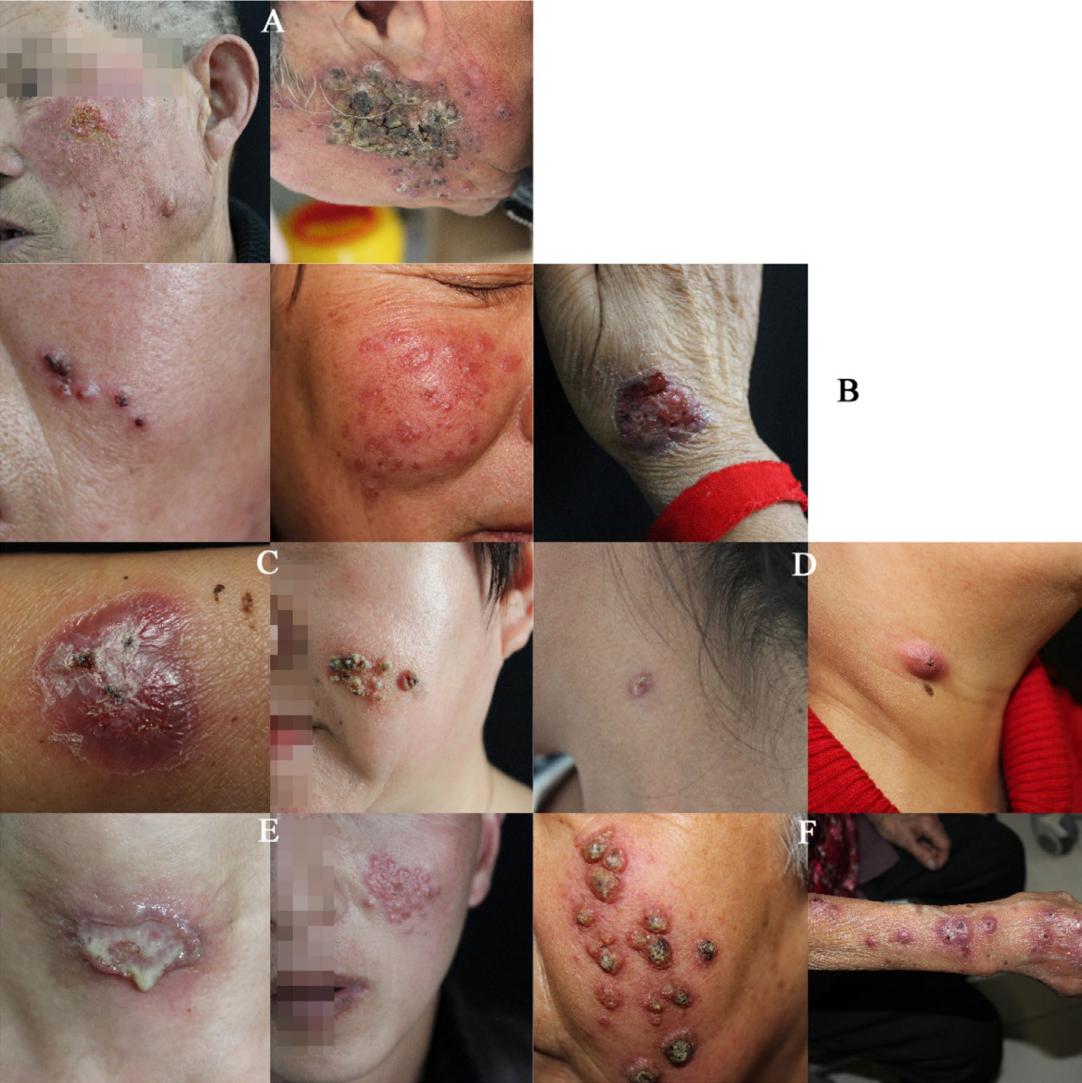
Pictures of the patient's skin lesions. (A) Verrucous type; (B) papular nodule type (left), ulcer type (right); (C) erythematous scales type (left), papulopustular type (right); (D) granulomatous type (left), erythematous type (right); (E) ulcers with pus (left), herpes zoster type (right); (F) lymphatic type on the face (left); lymphatic type on the upper limbs (right).

Among the 323 patients, 170 (52.7%) presented with the fixed type, 128 (40%) with the lymphatic type, 24 (7.00%) with the special type, and 1 (0.3%) with the cutaneous‐disseminated type; no cases of systemic type were observed. Within the fixed type category, there were 50 cases of squamous patch type, 36 cases of ulcer type, 26 cases of erythematous type, 8 cases of granulomatous type, 25 cases of papular nodule type, 23 cases of verrucous type, and 2 cases of papulopustular type. Among the special types, there were 18 cases of rosacea, 4 cases of acne, and 2 cases of herpes zoster. Clinical photographs illustrating various presentations of patients in this study are provided in Figure 1.

#### 3.2.2 Distribution of Skin Lesions

There were 146 patients with skin lesions on their faces, including 103 cases on the cheeks, 24 on the nose, 10 on the eyelids, 5 on the forehead, and 4 on the lips. Additionally, there were 12 patients with lesions on the lower jaw, 9 on the neck, 2 on both the face and neck, 1 each on the face and lower jaw, and 1 on the face and chest. Lesions on the trunk were observed in 9 patients, with 2 on the shoulder, 4 on the anterior chest, and 3 on the abdomen. Regarding the upper limbs, 140 patients had lesions: 52 on the right arm, 33 on the left arm, 35 on the right hand, 18 on the left hand, and 2 on both upper limbs. There was 1 patient each with lesions on the feet, legs, hands, lower limbs, and back. Among these cases, there were 78 cases of fixed type and 42 cases of lymphatic type (including 2 cases on the nose) skin lesions on the face. On the lower jaw, there were 8 cases of fixed type and 4 cases of lymphatic type. On the neck, there were 7 cases of fixed type and 2 cases of lymphatic type. On the body, there were 5 cases of fixed type and 4 cases of lymphatic type. On the upper limbs, there were 22 cases of fixed type and 65 cases of lymphatic type. Lastly, on the hands, there were 44 cases of fixed type and 9 cases of lymphatic type.

In this study, there were 20 children under 14 years old, among whom 19 had fixed types and 1 had lymphatic type. Statistical analysis revealed significant differences in clinical types and disease onset sites. Clinical sites were categorized into fixed types (97 on face‐neck‐lower jaw, 22 on upper limbs, 44 on hands, 5 on the trunk) and lymphatic type (50 on face and neck, 65 on upper limbs, 9 on hands, 4 on the trunk), with a statistically significant difference (*p* < 0.001).

#### 3.2.3 Clinical Types and Disease Course

The relationship between fixed and lymphatic sporotrichosis and the duration of the disease was analyzed, as detailed in Table 2.

**TABLE 2:** The correlation between various clinical types and the course of the disease.

Disease course (months/years)/Types	Fixed‐type	Lymphatic type
≤ 3 months	76	64
3 to ≤ 6 months	54	42
6 to ≤ 9 months	6	7
9 to ≤ 1 year	27	13
> 1 year	7	3

*Note: p* = 0.329, *p* > 0.05, no statistical significance.

### 3.3 Fungal Culture

All 323 patients underwent fungal cultures, resulting in 284 positive results, 17 cases with no fungal growth observed, and 22 cases with contaminated specimens.

### 3.4 Histopathological Analysis of Skin Lesions

**FIGURE 2:**
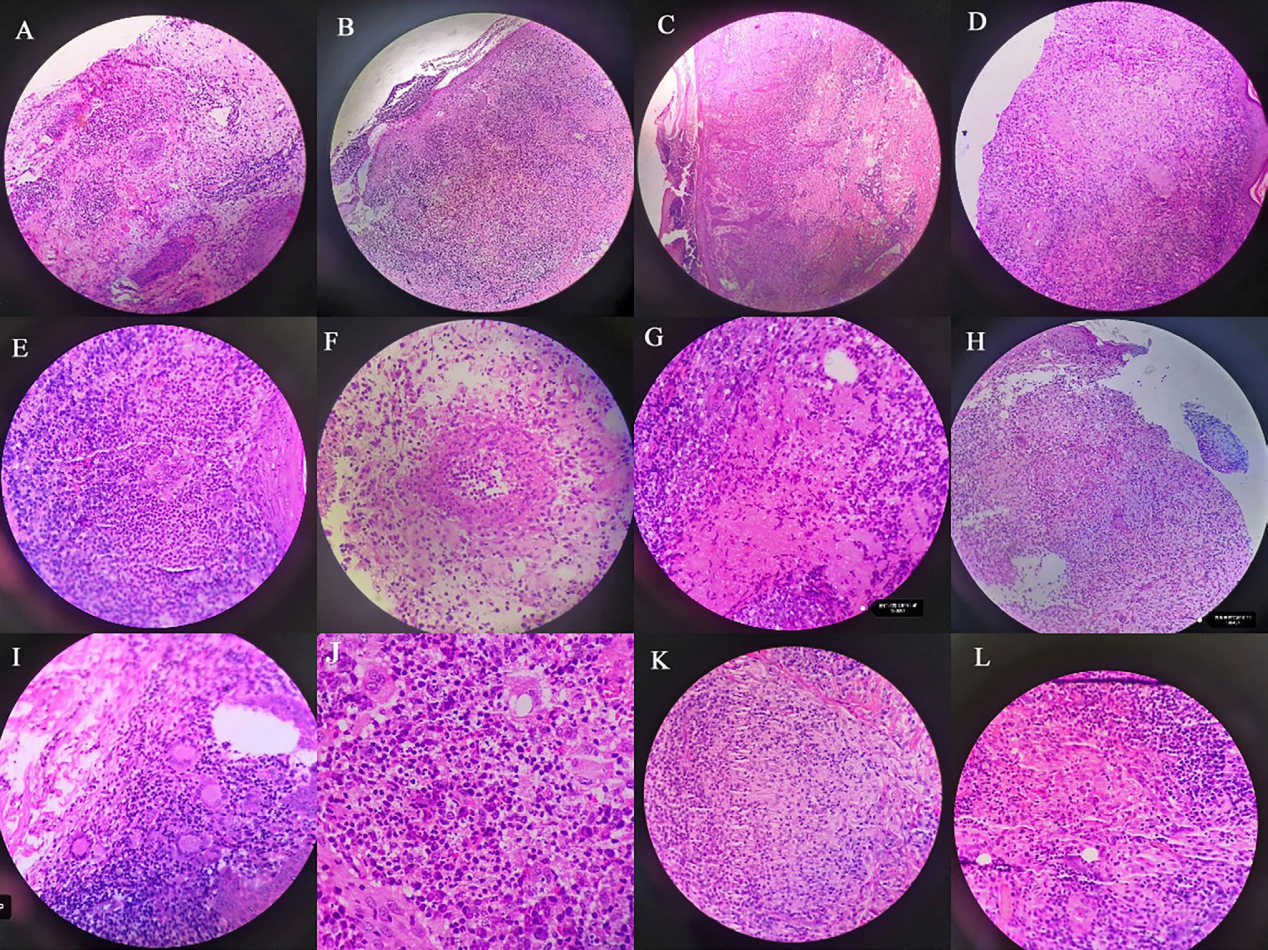
Representative pathological images. (A) Diffuse distribution (HE staining ×100); (B) surrounding tissue of vessels and appendages (HE ×400); (C) banding distribution of inflammatory cells (HE staining ×100); (D) tuberculoid granuloma (HE staining ×100); (E) purulent granuloma, HE staining ×400; (F) granuloma with three‐zone structure, HE staining ×400; (G) palisading granuloma, HE staining ×400; (H) sarcomatoid granuloma, HE staining ×400; (I) foreign body granuloma, HE staining ×400; (J) diffuse infiltration of each inflammatory cells, HE staining ×400; (K) well‐formed granuloma, HE staining ×400; (L) poorly formed granuloma, HE staining ×400.

Some pathological changes observed are detailed in Table 3, with representative pathological images depicted in Figure 2.

**TABLE 3:** Histopathological changes in 323 cases of sporotrichosis.

	Present^a^	Dominant^b^
Granuloma types		
Purulent granuloma	96 (30.0%)	61 (18.9%)
Tuberculoid granuloma	78 (24.1%)	64 (19.8%)
Granuloma with three‐zone structure	46 (14.2%)	22 (6.85)
Sarcomatoid granuloma	14 (4.3%)	4 (1.2%)
Palisading granuloma	8 (2.4%)	3 (0.9%)
Foreign body granuloma	5 (1.5%)	1 (0.3%)
Total	—	155 (48.0%)
Mononuclear phagocyte system (MPS)		
Epitheloid macrophages	285 (88.2%)	183 (56.7%)
Macrophages	226 (70.0%)	118 (36.5%)
Foam‐like cells	19 (5.9%)	9 (2.7%)
Multinuclear giant cells	232 (71.8%)	0 (0%)
Foreign body giant cells	5 (1.5%)	0 (0%)
Total	—	313 (96.9%)
Each inflammatory infiltration cells		
MPS	313 (96.9%)	91 (28.2%)
Lymphocytes	323 (100%)	114 (35.3%)
Plasma cells	283 (87.6%)	64 (19.8%)
Neutrophils	270 (83.6%)	54 (16.7%)
Eosinophils	36 (11.1%)	0 (0%)
None or rare	0 (0%)	0 (0%)
Total		323 (100%)
Necrosis types		
Liquefaction necrosis	92 (28.5%)	76 (23.5%)
Caseous necrosis	62 (19.2%)	34 (10.5%)
Coagulation necrosis	7 (2.2%)	2 (0.6%)
None	211 (65.4%)	211 (65.4%)
Total		323 (100%)

^a^ Pathological observation of pathological changes present in.

^b^ Athological observation of pathological changes mainly manifests as.

Up to three different types of granulomas were observed in the same sample: one type in 130 samples, two types in 24 samples, and three types in one case. Regarding necrosis, liquefactive necrosis and caseous necrosis were more common, and multiple types of necrosis could be observed in the same sample.

Tuberculoid granulomas and purulent granulomas were the predominant types of granulomas, with granulomas displaying three‐zone structures following. Other types of granulomas were rarely seen.

### 3.5 Treatment and Prognosis

Of the 185 patients who underwent follow‐up visits, 166 were completely cured while 19 were not cured, resulting in a cure rate of 89.7%. Most patients received treatment involving antifungal therapy like potassium iodide, itraconazole, terbinafine, fluconazole, or a combination of these drugs, as outlined in Table 4.

**TABLE 4:** Treatment and outcome of 185 cases of sporotrichosis.

Therapeutic medications	Number of cured cases	Number of uncured cases	Cured time (months)
Potassium iodide	17	0	2.26 ± 1.41
Itraconazole	52	6	3.29 ± 2.57
Terbinafine	27	2	3.11 ± 3.41
External use of itraconazole and terbinafine	1	2	—
Combined medication (itraconazole + KI/itraconazole + terbinafine/itraconazole + fluconazole)	61	8	3.16 ± 2.07
Traditional chinese medicines	2	1	—
Surgical resection	1	0	—
Folk prescription	5	0	—

## 4 Discussion

### 4.1 General Condition of Sporotrichosis

#### 4.1.1 Sex and Age of Disease Onset

This study indicates a significant predominance of female patients, particularly among farmers and domestic workers, potentially linked to local lifestyles. In rural Northeast China, women traditionally handle tasks like burning straw for heating and cooking, thereby increasing their exposure risk. This finding aligns with recent epidemiological data on sporotrichosis in Northeast China [7]. Similarly, studies conducted in India and Japan also highlight a higher prevalence among females, contrasting with many other countries where males engaged in labor‐intensive occupations are more susceptible to infection [8]. The disease predominantly affects individuals aged 41–67 years, primarily attributed to the occupational dynamics within rural settings where younger individuals often migrate for work, leaving middle‐aged and elderly individuals engaged in agricultural activities at home. Additionally, age‐related declines in immunity among the middle‐aged and elderly population may contribute to their increased susceptibility when compared with the young.

#### 4.1.2 Occupation and Traumatic History

*Sporothrix* organisms typically reside in soil, wood, and plants, primarily transmitted through skin, mucous membranes, upper respiratory tract, or digestive tract injuries. Therefore, the disease often correlates with occupational exposures. Among the 185 patients who underwent follow‐up visits, 141 (76.2%) were farmers, whose susceptibility stems from frequent contact with soil and organic matter. In recent years, there has been a rise in reports of zoonotic sporotrichosis, notably large‐scale outbreaks linked to cat scratches and bites in Brazil [9]. Conversely, there are few reports in China, possibly due to the prevalence of *Sporothrix globosa* locally, whereas the more pathogenic *Sporothrix brasiliensis* is predominant in Brazil and other regions [10].

#### 4.1.3 Course of Disease and Time of Disease Onset

In this study, 228 patients (70.4%) exhibited a disease duration of < 6 months, consistent with findings reported by Jiandong Zhang [11]. One patient had a disease duration of up to 5 years, possibly attributed to poor living conditions in rural areas and initial neglect of early disease symptoms. All 20 children (< 14 years old) had a disease duration of < 3 months, indicating heightened parental awareness and prompt medical intervention for pediatric cases. Regarding consultation times, patients with lymphatic type sporotrichosis sought medical attention sooner than those with fixed type, possibly due to the more severe clinical manifestations associated with lymphatic involvement, although this difference was not statistically significant (*p* > 0.05).

The incidence of sporotrichosis was significantly higher in winter and spring compared to summer and autumn (*p* < 0.05), indicating the influence of geography and climate on *Sporothrix* growth. In the summer months in this region, characterized by high temperatures, heavy rainfall, and low air pressure, it is hypothesized that these environmental conditions may inhibit the growth and reproduction of *Sporothrix*, thereby reducing its pathogenicity. Conversely, despite lower temperatures in winter, the enclosed environments associated with storing firewood and grass may provide favorable conditions for parasite growth, necessitating further investigation [12]. Additionally, in spring, agricultural activities like burning straw, plowing, and farming increase the exposure of farmers to pathogens, contributing to a higher prevalence rate, consistent with recent epidemiological trends of sporotrichosis in Northeast China [11].

### 4.2 Clinical Manifestations

#### 4.2.1 Clinical Manifestations of Sporotrichosis

The clinical manifestations of sporotrichosis are diverse, presenting as papules, erythematous scales, ulcers, abscesses, granulomas, verrucous hyperplasia, pustules, and more. Misdiagnoses have been reported, where sporotrichosis was mistaken for conditions such as prurigo nodularis, infectious granuloma, cutaneous tuberculosis, lichen planus, furuncles, erythema nodosum, impetigo, pityriasis rosea, chestnut lupus, and traumatic ulcers [13, 14, 15]. The lymphatic type of sporotrichosis displays rather typical symptoms that facilitate diagnosis, though it must be differentiated from conditions like pool granuloma. Disseminated sporotrichosis should be distinguished from diseases like vasculitis, tuberculosis, leprosy, chromoblastomycosis, paracoccidioidomycosis, and syphilis.

Skin lesions were predominantly observed on the face, neck, lower jaw, upper limbs, and hands, which are frequently exposed and susceptible to injury, with the face being the most common site (45.2%). The fixed and special types primarily manifested on the face, while the lymphatic type was more commonly seen on the upper limbs and face. There were statistically significant differences between the various clinical types and the locations of the lesions (*p* < 0.05).

#### 4.2.2 Clinical Classifications

Among the 323 samples in this study, there were 170 cases (52.7%) of the fixed type, 128 cases (40%) of the lymphatic type, 24 cases (7%) of the special type, 1 case of the cutaneous‐disseminated type, and no systemic type. This distribution is consistent with the 41.58% of lymphatic type and 55.14% of fixed type observed in 434 patients in Jilin Province as reported by Song Yang, but differs from the 23% of fixed type and 75% of lymphatic type in 119 patients in Rio de Janeiro as reported by Marcia Ramos‐e‐Silva [7, 16]. Zhao Bian proposes that in endemic areas of sporotrichosis, the varying immunity of individuals may prevent the spread of skin lesions to distant sites, leading to regional differences in clinical manifestations of the disease [2]. Additionally, variations in the toxicity of strains and different inoculation sites may result in diverse clinical types [17]. Among the 20 children in this study, 19 cases (95%) were of the fixed type and 1 case (5%) was of the lymphatic type, all presenting on the face with early skin lesions such as papules, granulomas, and erythematous scales. Sporotrichosis in children often involves the face, with the fixed type being more common [18].

### 4.3 Histopathology

#### 4.3.1 Epidermal Changes

Epidermal changes in early lesions of sporotrichosis are generally subtle and nonspecific. As the disease progresses, depending on the location and nature of the lesion, it may present with alterations such as changes in epidermal thickness, ulceration, acanthosis, pseudo‐epitheliomatous hyperplasia, hyperkeratosis, and parakeratosis. A high proportion of pseudo‐epitheliomatous hyperplasia, consistent with the findings of Quintella, L.P. was observed in this study [19].

#### 4.3.2 Dermal Changes

The dermis primarily exhibits diffuse infectious granulomas. In this study, inflammatory infiltration in sporotrichosis was mainly diffuse (*n* = 225, 69.8%) and non‐specific (*n* = 168, 52.0%). These findings align with both domestic and international reports [11, 19]. Non‐specific mixed inflammatory cell infiltration (without granulomas) accounted for the largest proportion (*n* = 168), potentially due to sampling from new or shallow skin lesions that were not fully developed. Among the 155 cases with granuloma structures, a single type of granuloma was observed in 130 samples, two types in 24 samples, and three types in one sample, indicating the possibility of observing multiple different types of granulomas in the same pathological specimen. The predominant granuloma structures in sporotrichosis were tuberculoid granulomas and purulent granulomas, followed by granulomas with a three‐zone structure, with other types being rarely observed. It is noteworthy that the pathological “three‐zone structure” is uncommon, with atypical structures not very clear and varying numbers of primary cells. The typical three‐zone structure can be observed in early‐stage inflammatory nodules. Lesions may extend through the entire dermis and subcutaneous tissue, exhibiting dermal edema, small vessel hyperplasia, dilation, congestion, red blood cell extravasation, and liquefaction and coagulation necrosis. Intravascular thrombosis, blood vessels filled with neutrophils, and increased fibroblast numbers with marked fibrosis in the upper dermis can be seen in cases with a prolonged disease course.

The initial pathological manifestations of sporotrichosis include an increase in neutrophils and fungal burden, which decreases with the appearance of granulomas within 28 days. This is accompanied by an increase in lymphocytes and plasma cells [6]. In this study, the following observations were made: (1) inflammatory cell infiltration was predominantly neutrophilic, with 95% of cases revealing diffuse infiltration and 90% exhibiting poorly formed granulomas; and (2) neutrophil dominance in granulomas composed of macrophages (*n* = 44) was significantly greater than that in granulomas composed of epithelioid cells (*n* = 10). This indicates that in sporotrichosis, granulomas primarily composed of neutrophils and macrophages are present in the early and acute stages, whereas mature granulomas formed by epithelioid cells are observed in the later stages of the disease. These findings are consistent with other experimental studies [20].

The mononuclear phagocyte system (MPS) is present in almost all cases, typically distributed in bands, patches, and nodules. Lymphocytes are found in 100% of cases, being the most widely distributed and dominant inflammatory cells. Plasma cells are present in 89% of cases, generally densely distributed in patches. Neutrophils are present in 83.6% of cases, forming small abscesses in the epidermis. In purulent cases, neutrophil necrosis and nuclear dust can be observed, indicating that the significant increase of lymphocytes, plasma cells, and neutrophils, along with the increase in MPS, is characteristic of the disease. These findings are consistent with numerous reports both domestically and internationally [19, 21].

### 4.4 Treatment and Prevention

Susceptibility reports from various regions indicate that *Sporothrix schenckii* , *Sporothrix globosa*, and *Sporothrix brasiliensis* are particularly susceptible to terbinafine [22]. This study showed that oral administration of potassium iodide and terbinafine resulted in a favorable response, with the shortest cure time. Approximately 60% of patients were cured after 3 months of combined therapy. A small number of patients experienced successful outcomes through surgical resection or traditional Chinese medicine. Treatment efficacy may vary based on factors like medication type, age, immune status, and individual susceptibility and resistance to the drugs, warranting further research. Although sporotrichosis can be cured, the course of treatment is prolonged, and the side effects of the medications are significant. Efforts should be made to promote and educate high‐risk susceptible groups about the disease, encouraging the use of protective gloves during work, trauma avoidance, and timely medical treatment in case of infection. Additionally, it is crucial to enhance the understanding and treatment of the disease among grassroots doctors. The development of new antimicrobials is also essential.

## 5 Conclusion

In this region, sporotrichosis predominantly affects females more than males and is mainly seen in middle‐aged and elderly individuals aged 41–67 years. The incidence is higher in winter and spring compared to summer and autumn. Fixed types are more prevalent than lymphatic types, with skin lesions primarily involving the face and neck. Histopathologically, sporotrichosis manifests as diffuse infectious granulomas, with inflammatory infiltration predominantly characterized by diffuse and non‐specific mixed inflammatory cell infiltration (without granuloma formation). The granulomas observed in sporotrichosis are mainly tuberculoid and purulent, followed by those with a three‐zone structure. A notable increase in lymphocytes, plasma cells, and neutrophils is characteristic of the disease, along with the increase of MPS cells.

## Ethics Statement

This study was conducted in accordance with the declaration of Helsinki. This study was conducted with approval from the Ethics Committee of The Second Hospital of Harbin Medical University.

## Consent

Consent for publication was obtained from every individual whose data are included in this manuscript. A written informed consent was obtained from all participants.

## Conflicts of Interest

The authors declare no conflicts of interest.

## Data Availability

The data that support the findings of this study are available from the corresponding author upon reasonable request.
